# Practical Points

**Published:** 1899-07-22

**Authors:** 


### PRACTICAL POINTS.

*Cottage Hospitals.—Can you give me some idea of the relative cost of erection of a cottage hospital of, say, eight or ten beds, and the cost of subsequent maintenance per bed? Also I have seen a little pamphlet giving useful information respecting the furnishing and fitting of cottage hospitals, but cannot trace it.—Middlesex.*

Your question you will find fully answered in Sir Henry Burdett's "Cottage Hospitals," published by the Scientific Press, 28, Southampton Street, Strand. There the cost of construction of several existing hospitals is given; the Maidenhead Cottage Hospital, for instance, is quoted as costing £1,925 to build and £350 for furniture, a total outlay of £2,275, this amount providing accommodation for eight beds. The relative cost for maintenance per bed occupied is given at £66 9s. 1d. The pamphlet you refer to is probably the one published by the Scientific Press, a republication from The Hospital of a list drawn up by the Hon. Sydney Holland some years ago, the only one we know of.

*Floors.—What kind of floors are considered the best for the wards of an isolation hospital?—Hospital Secretary.*

No substance is better for this purpose than terrazzo, which is now in great favour amongst hospital authorities. It lends itself to cleansing and disinfecting well, and in practice the one objection raised against its use, that it is cold and hard, is not found to seriously counterbalance its obvious advantages. Its great drawback is its tendency to crack if the slightest sinking of the walls takes place. A well-laid teak or oak floor is very good, however, and has a pleasanter effect no doubt, but taken all round nothing is better than terrazzo for the floors of a fever hospital.

*Number of Patients.—"Secretary" writes: Having adopted the uniform system of accounts and statistics, the following question has arisen, and I shall be glad of your statement as to the general practice in regard thereto. In some hospitals an in-patient after a certain period is, for statistical purposes, counted as a readmission or new case. Thus, at one hospital a patient after three months is treated, for statistical purposes, as a new patient. Hitherto our practice has been to reckon each patient, however long an inmate, as one case. But in one or two cases, where repeated operations have been necessary, patients have remained considerably over twelve months, and this materially affects our averages and makes comparison difficult.*

The point you raise has led to much abuse in unscrupulous hands. It is quite clear that one patient cannot by any process be turned into two, and it would be highly improper and indefensible to treat the same patient as more than one patient because he happened to reside above a certain number of weeks or months in an institution. The proper plan to pursue in the case of patients who have long residence in the hospital is to set forth prominently in the report the average number of days each patient is resident, the average number of beds occupied throughout the year, and then to add particulars of any such special cases as those you name where there has been prolonged residence owing to special causes, arising from the nature of the ailment from which the patient was suffering. The only safe plan is to provide that new patients only shall be treated as new cases, no matter how long residence an individual case may require to effect a cure.

